# Towards Multitarget Testing in Molecular Microbiology

**DOI:** 10.1155/2013/121057

**Published:** 2013-11-25

**Authors:** Deborah Steensels, Anne Vankeerberghen, Hans De Beenhouwer

**Affiliations:** Department of Microbiology OLV Hospital Aalst, Moorselbaan 164, 9300 Aalst, Belgium

## Abstract

Advantages of PCR assays over more conventional culture-based diagnostics include significantly higher sensitivities and shorter turnaround times. They are particularly useful when patient treatment has already been initiated or for specimens that may contain microorganisms that are slow-growing, difficult to culture, or for which culture methods do not exist. However, due to genome variability, single target testing might lead to false-negative results. This paper focuses on examples from our own experiences and the literature to provide insight into the limitations of single target testing in molecular biology. Lessons learned from these experiences include the careful design of diagnostic assays, preferably multitargeted, the importance of investigating the incidence and epidemiology of infection in detail, the frequent participation in appropriate quality assurance schemes, and the importance of continuous attentiveness by investigators when confronted with inconsistent results. In conclusion, multitargeted testing in microbiological molecular assays should be a rule.

## 1. Introduction

The introduction of molecular methods has had a positive impact in many areas of diagnostic microbiology. These tests have been proven to be often more sensitive and specific than classical testing, and they are particularly useful for specimens that may contain fastidious, slow-growing, or unculturable microorganisms or when patient treatment has already been initiated. In addition, identification based on genetic traits is more objective than the interpretation of conventional phenotypic characteristics.

The development of a commercial or an in-house molecular assay begins with a review of the current literature. This provides information concerning the choices of target genes used in previous studies, potential specificity or sensitivity problems, and additional information of clinical importance (e.g., cutoff values). All known subtypes or other known sequence variants (mutations, insertions, deletions, etc.) of the pathogen should be included in the specificity testing if feasible [1]. Once an appropriate target is selected, primers and probes can be designed. 

However, due to genome variability, single target testing might lead to false-negative results. Indeed, many variants exist today, but not all variants are known, and new variants emerge constantly according to the ever present Darwin's evolution theory. 

This paper focuses on examples from our own experiences and the literature to provide insight into how single target testing might lead to false-negative results and the important lessons to take into account in the future.

## 2. Examples from Our Own Experience

In 1996, when our clinical laboratory was expanded with a molecular department, very few commercial assays existed. In-house tests were developed, using classical PCR at first (with gel electrophoresis), later followed by real-time PCR (Q-PCR). Since all our tests have the same workflow, we chose to continue with in-house testing rather than switching to commercial platforms. All molecular assays are extensively validated and the laboratory is ISO15189 accredited since September 2008. Nevertheless, we already have discovered quite a lot of cases of false-negative results with single target testing. 

We reported a case of vertebral spondylodiscitis caused by *Mycobacterium bovis*, where diagnosis was complicated because of the lack of IS*6110* [2]. Nucleic acid amplification tests (NAATs) have the potential to provide a rapid, sensitive, and specific diagnostic assay for *M. tuberculosis* complex in clinical specimens because they have a higher sensitivity than acid-fast staining and are much faster than culture. Most (commercial) assays are based on insertion sequence *6110 *(IS*6110*) because of specificity for *M. tuberculosis* complex and the multicopy number. However, the complete absence or the presence of only a few copies of this sequence has been reported in some strains, particularly those circulating in Southeast Asia. A large number of clinical isolates of *M. tuberculosis* complex from South India had either a single copy (40%) or no copy (4%) of IS*6110*, thus indicating the need to incorporate additional target sites for improved detection. Barani et al. have recently evaluated a dual target PCR diagnostic assay combining two primer pairs (IS*6110* and TRC_4_) with improved test sensitivity [3].

In our case, Ziehl-Neelsen stain of the biopsy was positive for acid-fast bacilli; however, PCR testing for *M. tuberculosis* complex based on IS*6110* was negative. After 5 weeks, acid-fast bacilli were grown only on Löwenstein-Jensen. PCR for *M. tuberculosis* complex based on IS*6110 *was again negative but 16S rDNA and rpoB sequencing showed a 100% homology with *M. tuberculosis* complex. A multiplex PCR based on the deletion of a 12.7 kb DNA fragment in the genome of *M. bovis* compared to *M. tuberculosis *identified the strain as *M. bovis*. To our knowledge, this was the first isolation of a *M. bovis* strain lacking IS*6110* to be reported in Europe [2]. 

The detection of human respiratory syncytial virus (hRSV) on respiratory samples by Q-PCR, targeting the nucleoprotein (NP-) gene, was initiated in 2004. The PCR technique proved to be more sensitive than the antigen test [4]. In the autumn of 2006, several hRSV antigen positive samples (Respistrip, Coris Bioconcept) that were negative by Q-PCR were found [5]. Sequencing analysis of the amplification product of 5 hRSV Ag positive Q-PCR negative hRSV variants showed identical sequences. They all had 4 mutations located in the binding site of the probe used in Q-PCR. BLAST-analysis of the NP-gene, which is thought to be one of the more conserved genes of hRSV, showed that the “new” hRSV variant sequence was not present in GenBank (November 2006). 

In August 2009, we received a sample for hepatitis B virus (HBV) PCR analysis. The patient had a strong positive serology for HBsAg; however, Q-PCR was negative. Agarose gel electrophoresis showed the presence of an amplification product, which indicated that the detection problem was linked to the probe. The sample was sent to another laboratory, where Q-PCR was positive for HBV with a high viral load. Sequence analysis was performed and confirmed a mutation of the probe binding site. The forward primer had 1 mismatch and the reverse primer had 2 mismatches, but these mismatches were situated in the center of the primers and were thought to have a minimal effect on binding, explaining why a Q-PCR product was apparent on electrophoresis. The MGB-probe showed 1 mismatch (c/t) at the fourth 5′ prime nucleotide. Since binding of the MGB-probe is very sensitive to mismatches and binding of the probe at the 5′ end is extremely important for the 5′-3′ exonuclease activity of Taq polymerase, this mutation could explain the false-negative result. A dual target PCR analysis was developed to avoid such cases in the future.

In the summer of 2010, the positivity rate of enterovirus in CSF was high. Some CSF samples, however, contained a high white blood count but were negative in bacterial culture and with enterovirus Q-PCR [6]. For one sample with high clinical suspicion, a second Q-PCR was performed with other primers but the same probe [7], generating a larger amplification product that contains the binding sites of the primers used in the original first Q-PCR. This second PCR was positive (CT 32.85). To investigate whether mutations in the primer binding sites of the first PCR were present, the amplification product of the second Q-PCR was sequenced. A mismatch at the 3′ end (A→G) of one of the primers of the first Q-PCR was found. BLAST-analysis (NCBI) revealed that this mutation was very rare since only 9 matches were found. As of that moment, both Q-PCR analyses are performed for every sample. 

During the influenza season 2012-2013, we found less influenza A (InfA; 31%) positive samples compared to those positive for influenza B (InfB; 69%). In national epidemiological data, the contrary was observed: more influenza A than B (data not published). To investigate the possible cause, several InfA positive samples, obtained from another laboratory, were analyzed with our in-house PCR. For InfA H3N2 positive samples, similar results were found, but samples positive for InfA H1N1 were only weekly positive or false negative with our in-house PCR. Subsequently, these samples were analyzed with primers recommended by the CDC [8], with correct results for both InfA H3N2 and H1N1. Cycle sequencing was performed on the RNA extract of the false-negative samples. A mismatch (c/t) in our in-house primer was found on position 3 at the 3′ end, which causes poor binding of the primer. The corresponding primer recommended by the CDC has the same mismatch on position 11, which has no influence on the binding of the primer. A new double target in-house PCR analysis was designed and validated.

## 3. Examples from the Literature

### 3.1. *Chlamydia trachomatis*

*C. trachomatis* strains carry a 7500 bp cryptic plasmid. This plasmid is present at 4–8 copies and, therefore, is an attractive target for *C. trachomatis* NAATs. In 2006, a new variant of *Chlamydia trachomatis* (nvCT) was identified by Ripa and Nilsson [9, 10]. They observed an unexpected 25% decrease in the *C. trachomatis* incidence in Halland County, Sweden. Consequently, they analyzed specimens with their routine diagnostic system (Abbott m2000 real-time PCR; Abbott Laboratories, IL, USA) in parallel with the Artus *C. trachomatis* PCR (ARTUS, Hamburg, Germany), which targets the genomic ompA gene and not the cryptic plasmid. Thirteen percent of the samples were identified to be positive with the Artus PCR only.

These samples contained a mutant strain (nvCT) characterised by a 377 bp deletion in ORF-1 of the multicopy cryptic plasmid, which includes the target region of both the Roche and Abbott *C. trachomatis* NAATs available at that time. The failure to detect nvCT by both Roche and Abbott tests resulted in thousands of failed diagnoses and generated false-negative reports, leaving many patients untreated [11].

The currently available new redesigned dual-target assay Abbott RealTime CT/NG (January 2008) targets another cryptic plasmid sequence in addition to the sequence affected by the nvCT deletion, and the Roche COBAS TaqMan CT v2.0 (June 2008) detects the chromosomal ompA gene in addition to the sequence affected by the nvCT deletion. Only sporadic cases have so far been reported outside the Nordic countries. However, current knowledge regarding the presence and prevalence of nvCT in other countries is limited due to the few studies, to the fact that many European laboratories can still not detect the nvCT, and to the fact that those that can detect nvCT do not use nvCT-specific or other distinguishing NAATs [12].

### 3.2. *Neisseria gonorrhoeae*

The gonococcal porA pseudogene is a popular target for in-house *Neisseria gonorrhoeae* PCR methods. A study by Whiley et al. [13] presents an *N. gonorrhoeae* porin A pseudogene (porA) PCR false-negative result caused by the presence of a meningococcal porA sequence presumably acquired through horizontal genetic exchange and recombination. For *N. gonorrhoeae*, the problem is exacerbated by the fact that the species comprises numerous subtypes that exhibit considerable sequence diversity as well as propensity to mutate. Notably, the distribution of subtypes can vary geographically, temporally, and between patient groups. So, the performance may vary between patient populations because of the presence of different subtypes; and secondly, as in this case, the performance within a given population can suddenly change either due to the importation of new strains or to the mutation of currently circulating strains.

### 3.3. *Neisseria meningitidis*

A number of culture-negative meningococcal disease cases have been observed in many countries due to the increasing use of preadmission antibiotics. Therefore, detection of meningococcal DNA by PCR is widely used for patients with suspected meningococcal meningitis and negative cerebrospinal fluid cultures. Cavrini et al. [14] reported multiple nucleotide substitutions in two isolates of *Neisseria meningitidis* serogroup C causing false-negative detection, with a real-time PCR targeting the ctrA gene. A similar problem was encountered by Jaton et al. [15] with a clinical isolate of *N. meningitidis* serogroup B not detected by their real-time PCR targeting the ctrA gene. 

### 3.4. JC Virus (JCV) (Human Polyomavirus)

Landry et al. published a case of a rapidly progressive, fatal neurologic illness in a young mother, whose cerebrospinal fluid (CSF) JCV DNA PCR at a reference laboratory was falsely negative. Ultimately, brain biopsy established the diagnosis of progressive multifocal leukoencephalopathy (PML). Repeat PCR testing of the same CSF targeting a different region of the genome yielded a highly positive result. Once again, this highlights that, due to genome variability, false-negative PCR results can be obtained despite high levels of virus [16]. 

### 3.5. Human Immunodeficiency Virus (HIV)

A discrepant observation of an undetectable viral load in an immunodeficient pregnant HIV-1-infected patient of African origin with no prior antiretroviral treatment was reported by Debyser et al. [17]. Although clinical progression was present in this patient with tuberculosis and a low CD4 cell count, viral load determinations with both Amplicor Monitor (Roche Diagnostics, Basel, Switzerland) and NASBA assays (Organon Teknika, Boxtel, The Netherlands) revealed no detectable RNA levels. The presence of HIV-1 RNA in the plasma of the patient was demonstrated by an in-house RT-PCR. Subsequent HIV-1 RNA quantification with the branched DNA method revealed a high viremia. DNA sequence analysis of the gag gene identified a subtype G HIV-1 strain (HIV-1BL). This illustrates that genetic diversity observed in HIV-1, in addition to potential shifts in virus populations induced by the selective pressure of antiretroviral therapies, may influence detection or viral load quantification when using “single primer pair” techniques. 

### 3.6. Influenza Virus

A loss of sensitivity for the detection of the seasonal H3N2 strain circulating in February 2011 was observed with the xTAG respiratory viral panel (Luminex Molecular Diagnostics, Toronto, Canada) FDA-cleared assay [18]. Absence of detection of H1 and H3 subtypes in samples with a positive matrix signal was initially presumed to be indicative of the pandemic subtype, as was the case in many laboratories using this combination of conserved and subtyping targets. However, this lack of H1/H3 signal was subsequently discerned to be a result of mutations in the H3N2 strain resulting in failure of detection. 

A new influenza B variant was discovered in Singapore in April 2011 during diagnostic testing of a 3-year-old boy with respiratory symptoms [19]. InfB virus was isolated from culture and confirmed by standard immunofluorescence testing but was not detected by the routine, in-house influenza screening RT-PCR assay that targets the nucleoprotein (NP) gene. Subsequent sequencing investigations demonstrated that several other published assays targeting NP could also fail to detect this novel variant.

In fact, due to continual drifts in the influenza virus genome and the high potential for reassortment, not only among different subtypes but also between different lineages of influenza viruses circulating at any given time, reliable diagnosis can be challenging. Even the use of conserved targets is not immune to drifts, with several studies showing accumulation of mutations in internal conserved genes of influenza virus within a few months into the H1N1 influenza A pandemic [20, 21]. 

## 4. Discussion

Advantages of PCR assays over more conventional culture-based diagnostics include significantly higher sensitivities and shorter turnaround times. They are particularly useful when patient treatment has already been initiated or for specimens that may contain microorganisms that are slow-growing, difficult to culture, or for which culture methods do not exist. Also, the interpretation of genotypic tests is often less subjective than the interpretation of conventional phenotypic characteristics which often depends on the experience of the laboratory technician. Additional valuable features of PCR-based assays include (i) the ability to test for several targets concurrently (and thereby provide type and subtype information), detect other microorganisms with overlapping seasonality, and detect coinfections; (ii) the ability to be implemented using automated and high-throughput platforms that have the potential for testing large sample numbers and requiring minimum technician time; and (iii) the ability to be adapted rapidly for detection of novel targets. 

Nevertheless, these numerous examples of false-negative results highlight potential problems associated with single target assays that are incapable of adequately identifying and/or quantifying genetically divergent strains. In addition, there is probably an underestimation of these false-negative results, since not all these cases are detected. In case of the Swedish variant of *Chlamydia trachomatis*, the discovery was based on a shift in epidemiological data. In other cases, it was a suggestive clinical context or a positive test result obtained with an alternative test that drove further investigation and detection of the false-negative result.

Because of these examples, the decision was made in our lab to gradually replace all single target tests by multitarget assays. Evidently, the selection of appropriate target genes is crucial. Highly conserved genes that are essential for microbial survival should be included. Possibly, genes with a high copy number are included to obtain an adequate sensitivity. Our assays undergo periodic evaluation, for example, BLAST-analysis, to screen for recently and currently circulating divergent strains. In addition, epidemiological data are compared on a regular basis with the national surveillance data to detect any changes in performance characteristics. 

In-house testing has some (theoretical) advantages in comparison to commercial assays. The cause of the problem can be identified faster since the test was designed by the user. For commercial assays, the design is not well known, and laboratories will first repeat testing (with a different lot of reagents for instance) before contacting the manufacturer. Another advantage of in-house testing is flexibility. When a false-negative result is discovered, one can “easily” switch to another target, or add an additional target. Indeed, an important attribute for any given diagnostic system is the ability to be adapted rapidly to provide specific detection of the new variant. Commercial assays need considerably more time to correct current shortcomings. In addition, only a few commercial assays are based on multiple targets for the detection of one organism. 

On the other hand, in-house development requires more expertise and resources. Moreover, not all laboratory-developed assays are as thoroughly validated by individual laboratories as required by official instances (e.g., FDA), and evaluation of a small number of strains and/or small number of clinical samples may fail to bring about important performance characteristics. Also, studies have demonstrated that there is significant variation in the ability of in-house assays among clinical laboratories to reliably detect infectious agents [22, 23]. Commercial assays, in contrast, are often validated on large numbers and in a multicenter setting.

Molecular diagnostic methods are routinely used to make clinical decisions based on when and how to treat a patient as well as to monitor the effectiveness of a therapeutic regime and identify any potential drug resistant strains that may impact the long-term treatment program. Therefore, confidence in the reliability of the result provided by the laboratory service to the clinician is essential for patient treatment. Hence, suitable quality assurance and quality control measures are important to ensure that the laboratory methods and service meet the necessary regulatory requirements both at the national and international levels.

Frequent participation in appropriate quality assurance schemes organized by independent institutions has repeatedly proven to be valuable external quality control measures. In addition to assessing the diagnostic performance (analytical sensitivity and specificity) of different assays used in individual laboratories, the statistical analysis of the results provides an actual snapshot on the technologies of the commercial or in-house NAATs used for the detection of a given pathogen among the participants. In this context, it is extremely important that all relevant circulating strains, sooner or later, should be included. To ensure correct diagnosis of organisms subject to great variation, such as for instance influenza virus, it would be useful to implement an annual distribution of a panel of circulating strains at the beginning of each season, organized by a reference laboratory and/or public authority. This might be a more important feature of external quality control schemes than the detection of low copy numbers of etiological agents, on which the emphasis is nowadays. 

Important to note is the advent of microarray analysis, which has the unprecedented potential to simultaneously detect and identify thousands of microbial genes. Although improvements are still needed to make the majority of microarray applications amenable to clinical microbiology laboratories, the future role of these robust technologies in diagnostic microbiology is indisputable [24].

## 5. Conclusions

Lessons learned from these experiences include the careful design of diagnostic assays, preferably multitargeted, and the importance of investigating the incidence and epidemiology of infection in detail, the frequent participation in appropriate quality assurance schemes, and the importance of continuous attentiveness by investigators when confronted with inconsistent results. At local level, it is necessary for diagnostic laboratories using molecular techniques to be constantly vigilant about the possibility of emerging variants that may decrease the sensitivity of their frontline screening assays. In addition, this report highlights the need for diagnostic laboratories to test every in-house or commercial assay on their own population of circulating strains. For laboratory-developed assays, primers and probes should be adapted accordingly, so as to minimize the risk of diagnostic misses.

For commercial assays, manufacturers should describe the region which is targeted as detailed as possible.

Moreover, from a public health perspective, it is essential for diagnostic and research laboratories worldwide to continuously update and share gene sequences of (possible novel) bacteria and viruses in public databases. This allows manufacturers and laboratories to develop and update diagnostic assays for new variants and the assessment of their sensitivity and specificity, that is, multiple-sequence alignment of the available uploaded sequences against primer/probe sequences.

Both clinicians and laboratories must recognize the limitations of PCR, since misleading results may have serious consequences. Laboratories should be alert to this possibility and encourage clinicians to provide feedback on missed diagnoses. If suspicion remains high, clinicians should communicate with the laboratory, and a PCR assay targeting a different area of the genome should be performed. Results, both positive and negative, should always be interpreted in the broader context of the circulating strains present in the area, level of clinical suspicion, severity of illness, and risk for complications in a patient with suspected infection.

There is probably an underestimation of missed diagnosis by molecular techniques due to genetic divergence because all living creatures are prone to evolution and even very conserved genes are prone to mutagenesis (Darwin never sleeps!). 

In conclusion, multitargeted testing in microbiological molecular assays should be a rule.
